# Bioconversion of Mixed Alkanes to Polyhydroxyalkanoate by *Pseudomonas resinovornas*: Upcycling of Pyrolysis Oil from Waste-Plastic

**DOI:** 10.3390/polym14132624

**Published:** 2022-06-28

**Authors:** Jong-Min Jeon, So-Jin Park, Ye-Seung Son, Yung-Hun Yang, Jeong-Jun Yoon

**Affiliations:** 1 Green & Sustainable Materials Research and Development Department, Korea Institute of Industrial Technology (KITECH), Cheonan 31056, Korea; j2pco@kitech.re.kr (J.-M.J.); p0215sj@kitech.re.kr (S.-J.P.); yaeseung@kitech.re.kr (Y.-S.S.); 2 School of Industrial technology, University of Science and Technology (UST), Daejeon 34113, Korea; 3 Department of Biological Engineering, College of Engineering, Konkuk University, Seoul 05029, Korea; seokor@konkuk.ac.kr

**Keywords:** polyhydroxyalkanoate, mcl-PHA, *Pseudomonas resinovorans*, mixed alkane

## Abstract

Polyhydroxyalkanoate (PHA) is a biodegradable plastic that can be used to replace petroleum-based plastic. In addition, as a medium-chain-length PHA (mcl-PHA), it can be used to provide elastomeric properties in specific applications. Because of these characteristics, recently, there has been much research on mcl-PHA production using inexpensive biomass materials as substrates. In this study, mcl-PHA producers were screened using alkanes (n-octane, n-decane, and n-dodecane) as sources of carbon. The amount of PHA produced by *Pseudomonas resinovorans* using sole n-octane, n-decane, or n-dodecane was 0.48 g/L, 0.27 g/L, or 0.07 g/L, respectively, while that produced using mixed alkane was 0.74 g/L. As a larger amount of PHA was produced using mixed alkane compared with sole alkane, a statistical mixture analysis was used to determine the optimal ratio of alkanes in the mixture. The optimal ratio predicted by the analysis was a medium with 9.15% n-octane, 6.44% n-decane, and 4.29% n-dodecane. In addition, through several concentration-specific experiments, the optimum concentrations of nitrogen and phosphorus for cell growth and maximum PHA production were determined as 0.05% and 1.0%, respectively. Finally, under the determined optimal conditions, 2.1 g/L of mcl-PHA and 60% PHA content were obtained using *P. resinovorans* in a 7 L fermenter.

## 1. Introduction

World plastic production has been increasing every year and reached 368 million tons in 2019 [1]. Among this plastic, 79% is dumped in landfills or the environment, where it takes from around 20 to 600 years to degrade [2]. In addition, the incineration of plastic waste causes serious emissions of greenhouse gases, which accelerates global warming and abnormal climate change [3]. According to the sixth assessment report of the Intergovernmental Panel on Climate Change (IPCC), the usage of petroleum-based plastic materials is still increasing, and the report warned that this will lead to irreversible climate change within around 10 years [4]. As a result, much attention is being paid to the development and use of alternative eco-friendly plastic materials, such as biodegradable plastic from renewable resources.

Polyhydroxyalkanoate (PHA) is a biodegradable plastic that is regarded as a source of alternative materials because it has similar physical properties to petroleum-based plastic [5]. PHA is biosynthesized and accumulated by many bacteria in their cytoplasm as carbon storage materials when they encounter harsh growth conditions in the presence of excess carbon sources, which are classified into two groups, based on the numbers of carbon atoms composed of monomers, with different material properties: short-chain-length PHA (scl-PHA) composed of monomers with 3 to 5 carbon atoms and medium-chain-length PHA (mcl-PHA) composed of 6 to 14 carbon atoms [6]. Among such materials, mcl-PHA has numerous industrial applications, such as coating materials, pressure-delicate glues, and polymer-binding agents in organic solvent-free paints, and also it can be used in a series of biomedical applications [7]. It is produced by PHA-accumulating bacteria such as *Pseudomonas* species, and it has various physicochemical properties depending on the monomer composition and ratio [8,9]. The overall cost of the PHA production process is still expensive; therefore, there have been many studies that have attempted to use various carbon materials such as organic waste, seaweed biomass, animal fat, chitin, and oil waste [5,10]. Among them, pyrolysis oil based on plastic waste also has the potential for use as a carbon source for the production of PHA by micro-organisms. Pyrolysis oil contains aliphatic and aromatic hydrocarbon compounds depending on the catalyst type, plastic waste type, and cracking conditions [11]. The aliphatic hydrocarbon compounds from pyrolysis of plastic waste are predominantly composed of olefins (C20+), and these can be converted to low-carbon alkane or alkene compounds via hydrocracking [12].

PHA production based on alkanes has been studied since the 1980s, and it was discovered that many hydrocarbon-degrading bacteria can degrade and utilize various alkanes as a carbon source to grow and accumulate PHA in vivo [13]. Most alkanes can be utilized through the β-oxidation pathway with conversion to the carboxylic acid formed by alkane monooxygenase and then utilized to acyl-CoA, which can be used as a monomer for PHA accumulation [14]. Therefore, numerous studies have demonstrated the production of PHA from single alkanes by various *Pseudomonas* species, and n-octane was shown to be an economical carbon source for the production of mcl-PHA by *P.* *oleovorans* [15]. Since then, the maturation of plastic-waste-based pyrolysis oil conversion technology has drawn attention to the use of organic resources containing large amounts of alkane compounds. However, its uses have been limited to applications such as heating oil due to problems such as the fact that it must be additionally purified with a single compound for use in the chemical process. In the case of conversion to biodegradable plastic materials, polyethylene (PE) pyrolysis wax contains a low level of n-octane, and it was demonstrated that *P. oleovorans* is not suitable for producing mcl-PHA from PE pyrolysis wax as a sole carbon source [16]. There are differences in the efficiency of conversion of various alkanes to PHA monomers depending on the affinity with polymerase, and the portion of each alkane in the monomer composition of the produced PHA may be different. Consequently, each alkane affects the monomer composition of the produced PHA, which is an important factor for determining its physical properties [16]. Therefore, if waste oil or plastic pyrolysis oil, which contain mixtures of alkanes, are used for PHA production, it is necessary to find and evaluate a suitable PHA-producing strain for application to the alkane mixture resources.

In this study, *Pseudomonas* species were evaluated for production of mcl-PHA using alkane mixtures containing n-octane, n-decane, and n-dodecane as a sole carbon source, and the optimal condition for increasing PHA production was determined. In addition, the relationship between the ratio of various alkanes and the composition of the produced PHA monomer was determined.

## 2. Materials and Methods

### 2.1. Micro-Organism and Culture Conditions

*P. fluorescens* (ATCC 42821), *P. putida* (ATCC 1751), *P. resinovorans* (ATCC 12498), and *P. stutzeri* (ATCC 1066) were used to screen for producers of mcl-PHA from alkanes as a carbon source. All the strains and cultures were incubated with a working volume of 50 mL in a 250 mL flask at 30 °C for 48 h, and the initial pH was set to 7. Cell growth was monitored by measuring optical density at 600 nm (OD_600_). All the strains used in this study were precultured in LB medium at 30 °C for 24 h; then, 1% (*v*/*v*) of cultured cells were used for inoculation for further study. All components were sterilized via autoclaving for at least 20 min at 121 °C. As a preculture, 1% (*v*/*v*) frozen stock was incubated overnight at 30 °C in a shaking incubator in 14 mL round bottom tubes with 5 mL of Luria–Bertani medium broth (LB) (Difco, Detroit, MI, USA). To screen for the optimal strain, each strain was cultured in glucose-free M9 media containing either 10% n-octane, 10% n-decane, or 10% n-dodecane as a sole carbon source at 30 °C for 48 h. To compare cell growth and mcl-PHA production, *P. resinovorans* was cultured in LB medium, cultured in glucose free M9 media with various carbon sources, including 2% (*w*/*w*) of glucose, 10% (*v*/*v*) of mixed alkane (n-octane, n-decane, and n-dodecane of the same volume), and 10% of each alkane. 

The amount of nitrogen and phosphorus needed to maximize PHA production was determined using various concentrations in the medium and the determined mixed alkane. The effect of nitrogen and phosphorus concentration (0 to 1.0% and 0 to 5%, respectively) on *P. resinovorans* growth, PHA accumulation, and its monomer composition was studied. Finally, *P. resinovorans* was cultured in a 7 L bioreactor (GF Fermentech, Cheongju, Korea) with a working volume of 3 L at the optimized culture condition. It was operated at 30 °C for 72 h, with a stirring speed of 300 rpm and 3 V/min of gas flow for aeration.

### 2.2. mcl-PHA Recovery from Biomass

Methyl ethyl ketone (MEK), methanol, and chloroform were used as extraction and purification solvents for mcl-PHA recovery. MEK was added to the lyophilized cell and the mixture maintained at 60 °C for 6 h in sealed screw-top test tubes. Then, the tubes were briefly vortexed and incubated at room temperature. After mixing, the tubes were centrifuged for 10 min at 2500× *g*. The supernatant was transferred to a sealed screw-top tube, and then 3 volumes of methanol were added to remove the lipids remaining in the solvent. The PHA was dissolved with chloroform by heating at 100 °C in a heat block for 4 h. The tubes were incubated at room temperature until the solvent evaporated.

### 2.3. Design of Experiments and Mixture Analysis

To develop a strategy for optimizing cell growth and PHA production, a mixture analysis model of three alkanes (n-octane, n-decane, and n-dodecane) was developed and populated using a standard mixture-analysis methodology and the Minitab V19 program. For the design of mixture-analysis experiments to populate the model, we used a simplex lattice method. The degree of the lattice for this mixture analysis was 2; therefore, the experimental design contained the set of all 10 combinations. All experiments were performed using 50 mL cultures with 20% total alkane content, and the cultures were cultured at 30 °C for 48 h in duplicate. To plot mixture contours, a mixture regression using the model-fitting method was applied with full quadratic component terms initially included. In the data analysis, the coefficients with p value below 0.1 were used as parameters.

### 2.4. Characterization of Obtained mcl-PHA

The quantity and composition of PHA were determined by gas chromatography (GC) and gas chromatography-mass spectrometry (GC-MS), using a slight modification of a method described previously [17]. For analysis, the microbial culture after the completion of growth was centrifuged at 10,000× *g* for 30 min, washed with deionized water two times, and suspended in 1 mL of water. The suspended samples were subjected to lyophilization, and the freeze-dried cells from each experiment were subjected to methanolysis. A weighed sample was placed in a Teflon-stoppered glass vial, and 1 mL chloroform and 1 mL methanol/sulfuric acid (85:15 *v*/*v*) were added to the vial. The samples were incubated at 100 °C for 2 h, cooled to room temperature, and incubated on ice for 10 min. After adding 1 mL of ice-cold water, the samples were mixed thoroughly using a vortex for 1 min and then centrifuged at 2000× *g*. The organic phase (bottom) was carefully extracted using a pipette and was moved to clean borosilicate glass tubes. A 2 µL portion of the organic phase of these samples was then injected into a gas chromatograph (6090N, Agilent Technologies, Santa Clara, CA, USA) using a flame ionization detector (FID) and a 30 m × 250 μm DB-FFAP capillary column with hydrogen as the carrier gas. The inlet of the gas chromatograph was maintained at 250 °C, and the oven was held at 80 °C for 5 min, heated to 220 °C at 20 °C min^−1^, and then held at 220 °C for 5 min. Peak detection was performed using a flame ionization detector, which was maintained at 300 °C. The fatty acid content was analyzed via GC–MS chromatography (Perkin Elmer Clarus 500, Waltham, MA, USA) according to the modified method previously reported in [17]. About 1 uL of methanolized sample was injected into the Clarus 680 GC-MS equipped with triple axis detector carrying Elite 5 ms column (30 mm length × 0.25 mm internal diameter × 0.25 mm film) at a split ratio of 10:1 with column flow 1.0 mL min^−1^. The injector temperature was set at 280 °C while the oven and column temperatures were programmed as 10 °C for 1 min, then increased to 130 °C at 11 °C min^−1^, held for 2 min, and increased to 310 °C at 10 °C min^−1^, and held for 10 min. Helium was used as carrier gas at 47.3 mL min^−1^ and 0.40 bar pressure. Mass spectra were acquired at 1250 scan speed using electron-impact energy of 70 eV at 200Uc ion source and 280 °C interface temperatures, respectively. Complete instrument control was available through TurboMass™ driver. NIST/EPA/NIH library was used to predict the methylated PHAs and their corresponding mass ion. Statistical analysis was carried out through one-way ANOVA, where *p* < 0.05 was considered to be statistically significant.

To study the melting behavior of synthesized polymer, differential scanning calorimetry (DSC) analysis were performed by Discovery DSC (TA Instruments, Bellefonte, PA, USA) in the temperature range from −80 to +100 °C. The glass transition temperature (Tg) was determined at a heating rate of 20 °C/min. In this study, Tg was taken as the midpoint of the step-transition. The weight-average molecular weight (Mw), number-average molecular weight (Mn), and polydispersity (Mw/Mn) were determined by gel permeation chromatography (GPC) conducted in THF solution at 35 °C and a flow rate of 1 mL/min. A 10 μL sample in THF at a concentration of 1% *w*/*v* was injected. Polystyrene standards with narrow molecular-mass distribution were used to generate a calibration curve.

## 3. Results and Discussion

### 3.1. Screening of Alkane-Based PHA-Producing Strains

**Figure 1:**
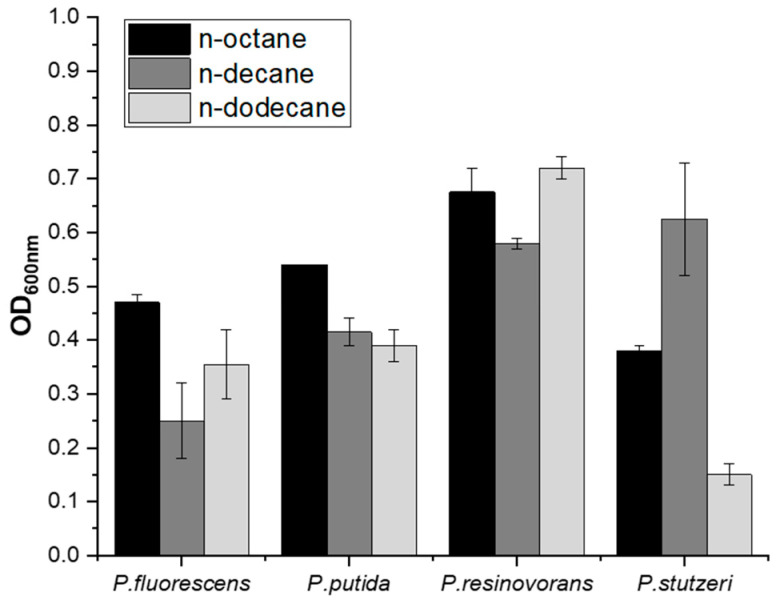
Cell growth of *Pseudomonas* species using n-octane, n-decane, and n-dodecane as a sole carbon source. Each alkane is present as 10% (*v*/*v*) in minimal medium.

**Figure 2:**
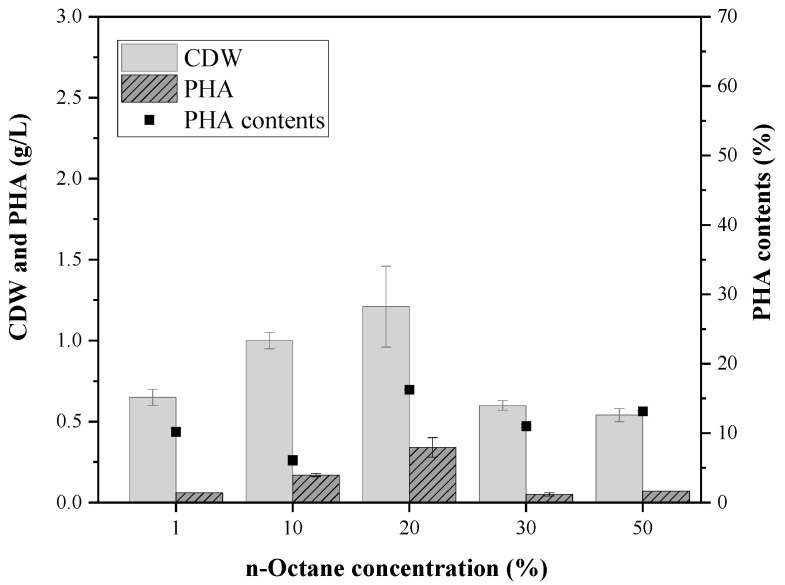
Cell growth and PHA production by *P. resinovorans* in the range of n-octane.

After the discovery in 1983 that *P. oleovorans* could produce mcl-PHA using n-octane, various *Pseudomonas* species were found to be capable of PHA production using alkane compounds [18,19,20]. However, few studies have reported on the use of various alkane compounds as a carbon source because most studies were focused on the use of n-octane. The composition of pyrolysis oil from polyethylene-based waste plastics is determined by its process conditions, and C8- to C22-saturated hydrocarbons are predominantly included in the case of pyrolysis oil received from the Korea Institute of Industrial Technology (Supplementary Figure S1). To find a suitable strain that is capable of mcl-PHA production using alkanes, *P. fluorescens*, *P. resinovorans*, *P. stutzeri*, and *P. putida* were selected and cultured in minimal media with 10 (*v*/*v*)% of either n-octane, n-decane, or n-dodecane as the sole carbon source. Each species showed different growth activity, and *P. resinovorans* showed better growth than the others (Figure 1). In addition, *P. resinovorans* was cultured in range of 1 to 50% of n-octane to investigate the relationship between the concentration of alkane and growth. The highest cell growth (1.34 g/L), PHA production (0.31 g/L), and PHA content (approximately 20 (*w*/*w*)%) were produced with 20 (*v*/*v*)% of n-octane (Figure 2). Meanwhile, with more than 30% n-octane in the medium, the cell dry weight (CDW), PHA amount, and PHA content decreased sharply to 0.68 g/L, 0.09 g/L, and 13.45%, respectively, which is attributed to the oxygen rate being rapidly reduced as the oil and liquid ratio increased [21].

*P. resinovorans* was selected for the recycling of pyrolysis oil, which contains various olefin compounds, because it is able to utilize alkanes and produces more mcl-PHA than the other *Pseudomonas* species, including *P. oleovorans*. In addition, to maximize the production of mcl-PHA, the optimum concentration of alkanes in the medium was determined to be 20%.

### 3.2. PHA Production by P. resinovorans Using Various Types of Carbon

Microbial metabolism for the accumulation of PHA occurs in two different ways: (1) short-chain carbon sources are usually utilized by biosynthesis for accumulation of PHA by the phaABC pathway, through acetyl-CoA to acetoacetyl-CoA by phaA (thiolase), while phaB (oxidoreductase) and phaC (polymerase) are mainly involved in intracellular processes; and (2) long-chain carbon sources, such as fatty acids or alkanes, are utilized through the β-oxidation pathway, and various acyl-CoA transferases convert to intermetabolites, including the mcl-PHA precursor [17]. *P. resinovorans* has both metabolisms for PHA accumulation; therefore, there is a need to evaluate the PHA production of different carbon sources. *P. resinovorans* was cultured using 2% of glucose, 20 (*v*/*v*)% of each alkane (n-octane, n-decane, or n-dodecane), and 20 (*v*/*v*)% of mixed alkane to compare PHA production and the monomer composition in accordance with the carbon source (Table 1). With glucose as the carbon source, 1.56 g/L of PHA was produced that contained 1.47% 3-hydroxyhexanoate (3HHx), 10.60% 3-hydroxyoctanoate (3HO), and 87% 3-hydroxyoctanoate (3HD). Among the carbon sources with 20% of a single alkane (n-octane/n-decane/n-docane), the best cell growth and PHA production were found using n-octane as the sole carbon source. This produced a CDW of 1.74 g/L, PHA of 0.37 g/L, and PHA content of 22.4% that contained 4.59% of 3HHx, 86.05% of 3HO, and 9.36% of 3HD. When the mixed alkane was used, 0.69 g/L of PHA was produced that contained 11.77% of 3HHx, 74.39% of 3HO, and 13.84% of 3HD. Although cell growth was not the highest, the mixed alkane showed an approximately 50% increase in PHA production compared with using n-octane.

### 3.3. Mixture Analysis of Alkanes (n-octane, n-decane, and n-dodecane) as a Carbon Source for mcl-PHA Production

**Figure 3:**
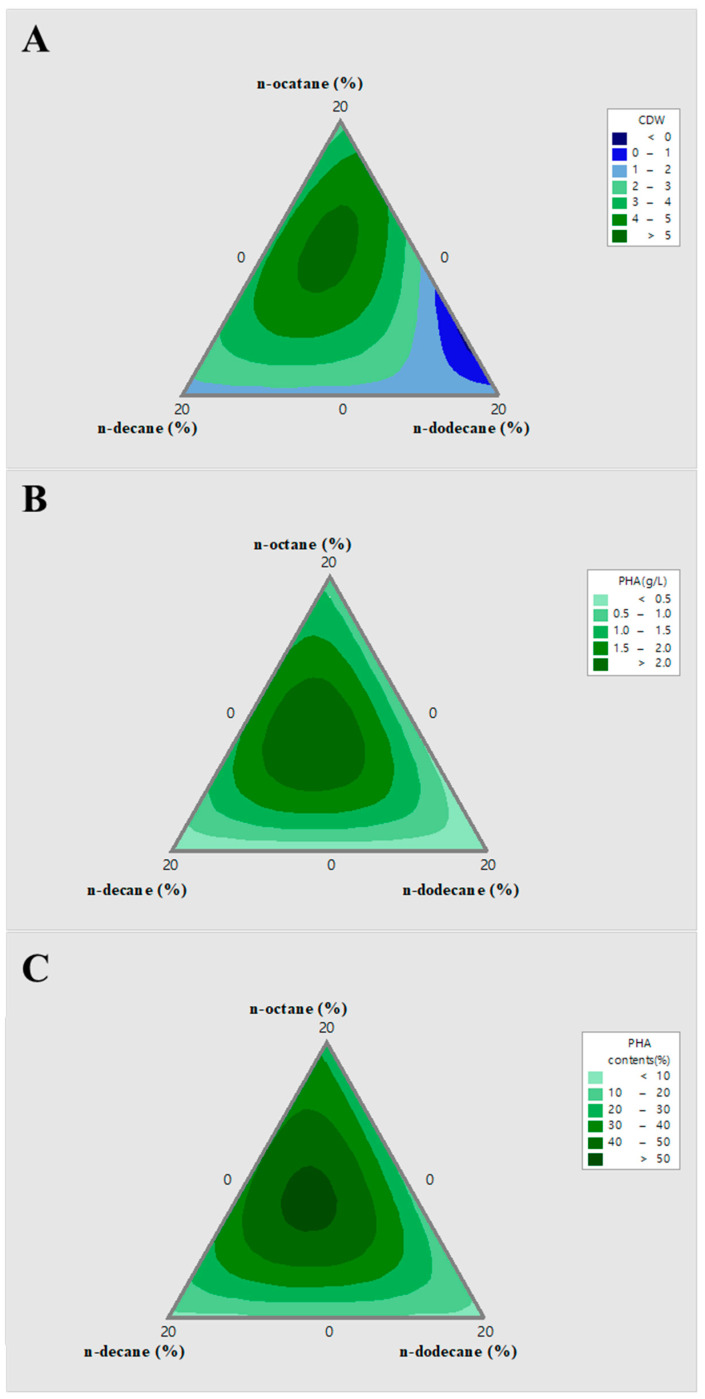
Mixture contour plots of mixed alkane composition for PHA production. The optimal composition ratio between n-octane, n-decane, and n-dodecane was determined as 20% (*v*/*v*) as a total concentration. (**A**): CDW (g/L), (**B**): PHA (g/L), and (**C**): PHA content (%) of different mixed alkane compositions.

To investigate the effect of the three alkanes on cell growth, PHA production, and PHA content, *P. resinovorans* was grown as 10 culture compositions based on the mixture analysis model, as described in “Materials and methods” (Table 2). For each culture, we measured the cell growth, amount of PHA, and PHA content. The results of the statistical analysis were shown by contour plots and the predicted highest cell growth, PHA titer, and PHA content using *P. resinovorans* were determined (Figure 3).

Contour plots for cell growth predicted the best ratio to be when n-octane, n-decane, and n-dodecane are 10.36%, 5.48%, and 4.5%, respectively, which produced CDW of 5.28 g/L (Figure 3A). In the case of 9.15% of n-octane, 6.44% of n-decane, and 4.29% of n-dodecane, the PHA titer was 2.39 g/L of PHA (Figure 3B). In addition, the PHA content calculated by the amount of PHA contained in the cell was predicted to be a maximum of 52.33% content (*w*/*v*) when n-octane, n-decane, and n-dodecane are 8.63%, 6.86%, and 4.50%, respectively (Figure 3C). The optimal alkane composition for total PHA production predicted by the model is a combination of n-octane, n-decane, and n-dodecane, rather than pure alkane or a combination of two different alkanes [16,22,23]. The reason why a combination of alkane compounds in the medium is beneficial is not currently clarified, but is estimated to be due to the better activities of alkane hydroxylase of *P. resinovorans* when the mixed alkane exists [24,25].

### 3.4. Jar-Scale Fermentation for mcl-PHA Production in Media-Optimized Conditions

**Figure 4:**
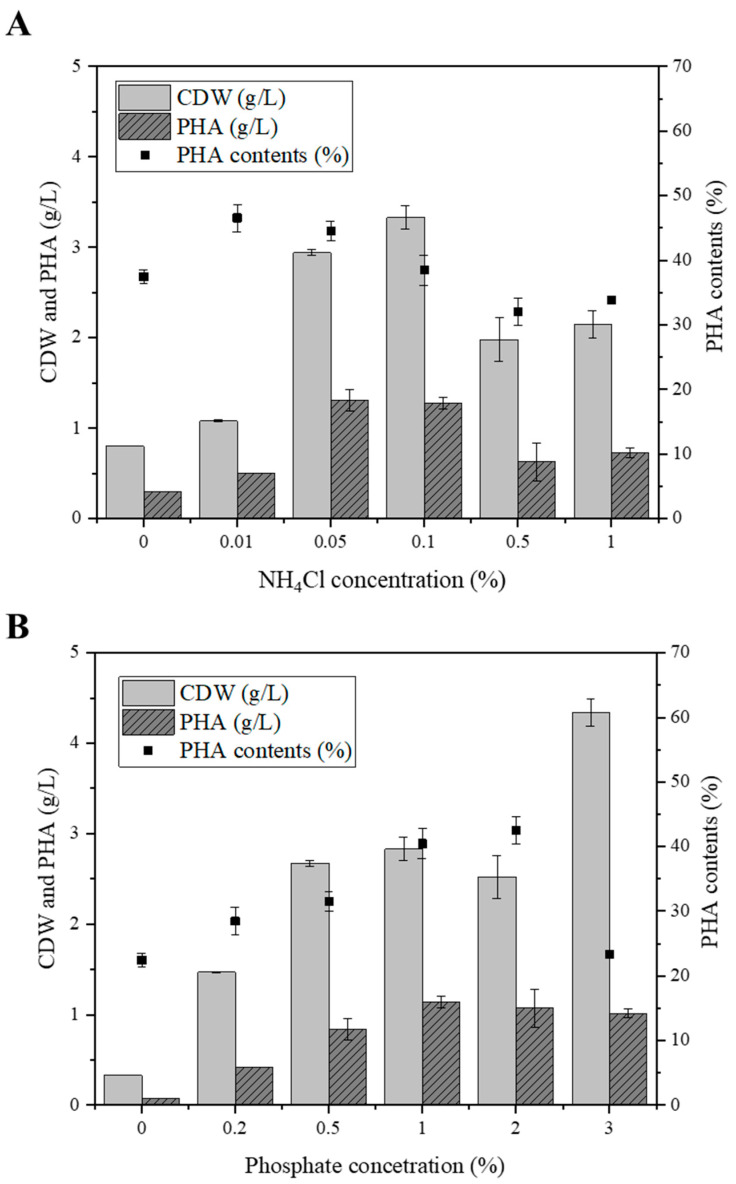
Effects of nitrogen and phosphorus limitation on PHA production. (**A**): CDW, PHA titer, and PHA content (%) in range of 0 to 1% nitrogen concentration. (**B**): CDW, PHA titer, and PHA content (%) in range of 0 to 3% phosphate concentration.

Carbon, nitrogen, and phosphorus are essential nutrients for growth, and their limitation can trigger mcl-PHA production in micro-organisms. However, the specific molecular mechanisms that drive this synthesis in *Pseudomonas* species under unfavorable growth conditions remain poorly understood. Therefore, it is necessary to determine the optimal concentration of nitrogen and phosphorus, because mcl-PHA production is related to their concentration when alkanes are used as carbon sources. To determine the optimal conditions, nitrogen concentrations were observed from 0% to 1.0% and phosphorus concentrations from 0% to 3.0% (Figure 4). When the concentration of nitrogen in the culture medium increased from 0 to 0.05%, PHA production tended to increase accordingly. The maximized PHA produced in 0.05% nitrogen was 1.31 g/L, but in the medium where the nitrogen concentration was more than 0.1%, PHA production decreased (Figure 4A). When the concentration of phosphorus in the medium increased from 0 to 1%, the production of PHA increased as well (Figure 4B). The maximum PHA production was 1.14 g/L at a concentration of 1% phosphorus. However, PHA production tended to decrease when the concentration of phosphorus was more than 2%. These results suggest that the optimal phosphorus concentration in the medium is 1.0%.

**Figure 5:**
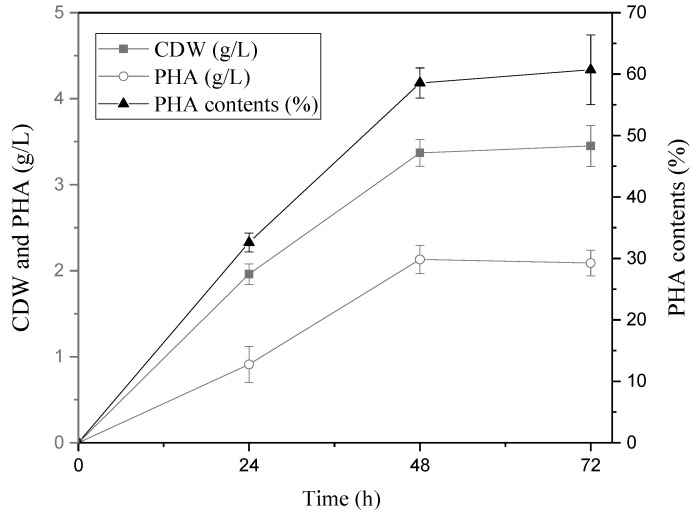
PHA production by *P. resinovorans* in optimized condition. Batch culture was performed in 7 L scale jar fermenter (3 L as working volume) with optimized culture medium.

Using the optimized alkane combination and nitrogen and phosphorus concentration, *P. resinovorans* was cultured in a 7 L jar fermenter (3 L of working volume). The CDW, amount of PHA, and PHA content reached 3.5 g/L, 2.1 g/L, and 60%, respectively (Figure 5). Previous reports focused on *P. oleovorans* for the production of mcl-PHA with n-octane, and these show production of up to 16.8 g/L of PHA by fed-batch culture, while n-decane and n-dodecane were not considered because of low PHA production (Table 3). Compared with other *Pseudomonas* species, it was proven that use of *P. resinovorans* is more effective for production of mcl-PHA when mixed alkane is used as a carbon source.

### 3.5. Physical Properties of Produced mcl-PHA by P. resinovorans

The thermal properties of the produced mcl-PHA were determined by means of DSC (Table 4 and Supplementary Figure S2). The Tg of the samples ranged from −43.44 to −34.29, which can be regarded as values typical for this type of PHA. The sample also showed a Tm in the range of 39.62 with a ΔHm of 13.2 J/g. In addition, these values are typical for mcl-PHAs and indicate classical rubber- to latex-like characteristics [26]. The Mw, Mn, and polydispersity (Mw/Mn) were similar for the copolyesters, despite the differences in their monomer compositions. The Mn and Mw were 267,649 and 630,526 Da, respectively.

In addition, Z average (Mz) and Z + 1 average (Mz + 1) molar mass were determined as 1,110,977 and 1,729,603 Da, respectively. The polydispersity index (PDI) was calculated (PDI = Mw/Mn) as 2.36, more or less similar to that of the PHA value of mcl-PHAs biosynthesized by other known *Pseudomonas* species.

## 4. Conclusions

Recent studies have shown that the use of renewable carbon sources can produce a significant reduction in actual production costs. A very interesting research topic would be for industrially produced waste oil or oil produced by pyrolysis of waste plastics to be used as a fermentation substrate for production of PHA, which is a promising bioplastic. Therefore, it is important to find PHA-producing micro-organisms and establish culture conditions in accordance with the carbon composition of waste oil or oil from waste plastics.

In this study, the ability of *P. resinovorans* to produce PHA from alkanes (n-octane, n-decane, and n-dodecane), especially mixed alkane, was identified, and a model was built to improve mcl-PHA production. Compared with other *Pseudomonas* species, *P. resinovorans* was much more effective in utilizing mixed alkane, and it can be an attractive microbial host for production of PHA. Although the regulation of PHA production based on alkane utilization has not been discovered, the results of this study are meaningful for use as basic data for the application of various renewable carbon resources containing alkanes.

## Figures and Tables

**Table 1:** Cell growth, PHA production, and monomer composition by *P. resinovorans* in various carbon sources.

Substrates	mol% of 3HHx	mol% of 3HO	mol% of 3HD	CDW (g/L)	PHA (g/L)	PHA Content (%)
2% of glucose	1.47 ± 0.03	10.60 ± 1.76	87.93 ± 3.22	2.68 ± 0.09	1.56 ± 0.04	57.76 ± 2.35
20% of mixed alkane	11.77 ± 0.26	74.39 ± 3.15	13.84 ± 2.12	1.46 ± 0.03	0.69 ± 0.09	47.46 ± 1.12
20% of n-octane	4.59 ± 0.12	86.05 ± 4.33	9.36 ± 0.06	1.74 ± 0.07	0.37 ± 0.01	22.35 ± 0.08
20% of n-decane	-	40.42 ± 1.43	61.18 ± 6.34	1.23 ± 0.11	0.24 ± 0.01	12.99 ± 0.06
20% of n-dodecane	-	-	97.50 ± 0.05	0.39 ± 0.09	0.08 ± 0.04	22.66 ± 0.02

3HHx: 3-hydroxyhexanoate, 3HO: 3-hydroxyoctanoate, 3HD: 3-hydroxydecanoate.

**Table 2:** Monomer composition of 10 conditions by mixture analysis.

ID #	n-octane (*v*/*v*%)	n-decane (*v*/*v*%)	n-dodecane (*v*/*v*%)	mol% of 3HHx	mol% of 3HO	mol% of 3HD	PHA (g/L)	PHA Content (%)
1	20	0	0	3.55	69.44	27.01	0.42	21.69
2	10	10	0	5.43	59.41	35.16	1.25	34.72
3	10	0	10	4.14	67.09	28.81	0.63	26.53
4	0	20	0	-	-	100	0.03	2.21
5	0	10	10	-	44.88	55.12	0.21	14.26
6	0	0	20	-	-	-	-	-
7	6.66	6.66	6.66	6.02	84.32	9.67	1.68	41.78
8	13.33	3.33	3.33	10.28	80.18	9.54	2.86	50.46
9	3.33	13.33	3.33	41.03	32.21	47.28	1.64	40.69
10	3.33	3.33	13.33	4.43	80.99	14.58	1.17	29.33

3HHx: 3-hydroxyhexanoate, 3HO: 3-hydroxyoctanoate, 3HD: 3-hydroxydecanoate.

**Table 3:** mcl-PHA production using various alkanes by Pseudomonas species.

Organism	Carbon Source	CDW	Amt of PHA	Monomer Composition	Cultivation Mode	Reference
*P. oleovorans*	n-octane	-	13.4 g/100 g of CDW	3HHx, 3HO	Batch	[22]
*P. oleovorans*	n-decane	-	5.1 g/100 g of CDW	3HO, 3HD	Batch	[22]
*P. oleovorans*	n-dodecane	-	-	-	Batch	[22]
*P. aeruginosa* GL-1	Pyrolysis oil (C8 to C27 alkane)	0.39 (g/L)	0.07 (g/L)	3HHx, 3HO, 3HN, 3HD, 3HUD, 3HDD, 3HTD	Batch	[16]
*P. oleovorans*	Pyrolysis oil (C8 to C27 alkane)	-	-	-		[16]
*P. resinovorans*	octanoic acid	4.6 (g/L)	0.4 (g/L)	3HB, 3HHx, 3HO, 3HD	Continuous	[23]
*P. resinovorans*	n-octane, n-decane and n-dodecane	3.5 (g/L)	2.1 (g/L)	3HHx, 3HO, 3HD	Batch	In this study

CDW: final cell dry weight, PHA formed: final concentration of PHA, 3HB: 3-hydroxybutyrate, 3HHx: 3-hydroxyhexanoate, 3HO: 3-hydroxyoctanoate, 3HN: 3-hydroxynonanoate, 3HD: 3-hydroxydecanoate, 3HUD: 3-hydroxyundecanoate, 3HDD: 3-hydroxydodecanoate and 3HTD: 3-hydroxytetradecanoate.

**Table 4:** Physical properties of mcl-PHA from *P. resinovorans* using mixed alkane.

T_g_ (°C)	T_m_ (°C)	ΔHm (J/g)	Mw (Da)	Mn (Da)	PDI
−38.9	48.2	13.2	267,649	630,526	2.36

## Supplementary Materials

The following supporting information can be downloaded at: https://www.mdpi.com/article/10.3390/polym14132624/s1, Figure S1: Pyrolysis oil of waste-plastic and its composition, Figure S2: DSC analysis of purified PHA produced by *P. resinovorans* using mixed alkane.
