# Supplementary material for: Bioconversion of Mixed Alkanes to Polyhydroxyalkanoate by Pseudomonas resinovornas: Upcycling of Pyrolysis Oil from Waste-Plastic
Source: Polymers (Basel). 2022 Jun 28;14(13):2624. doi: 10.3390/polym14132624 (PMC9268961; doi:10.3390/polym14132624)

Bioconversion of mixed alkanes to polyhydroxyalkanoate (PHA) by *Pseudomonas resinovornas*: Upcycling of pyrolysis oil from waste-plastic

Jong-Min Jeon[a], So-Jin Park[a,b], Ye-seung Son[a], Yung-Hun Yang[c], and Jeong-Jun Yoon[a]

[a] *Green & Sustainable Materials R&D Department, Korea Institute of Industrial Technology (KITECH), Republic of Korea*

[b] *School of Industrial technology, University of Science and Technology (UST), Republic of Korea*

[c] *Department of Biological Engineering, Konkuk University, Republic of Korea*

* Corresponding author. Green & Sustainable Materials R&D Department, Korea Institute of Industrial Technology (KITECH), 89 Yangdaegiro-gil, Ipjang-myun, Seobuk-gu, Cheonan-si, Chungnam, 330-825, Republic of Korea

E-mail address: jjyoon@kitech.re.kr

Office number: +82-41-589-8266

Supplementary Figure S1. Pyrolysis oil of waste-plastic and its composition. A: Pyrolysis oil of commercial polyethylene waste (yellow) and purified with zeolite as catalyst (red), B: purified pyrolysis oil composition.

A

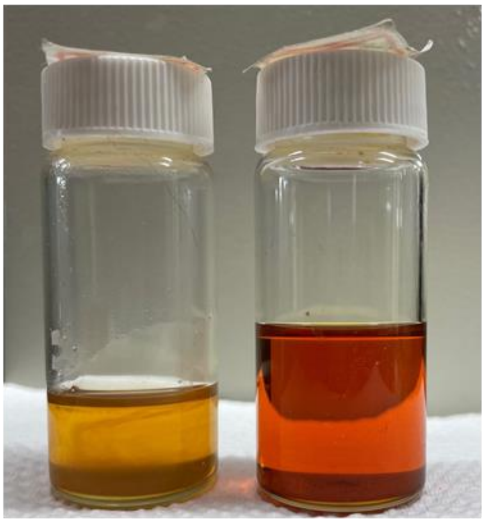

B

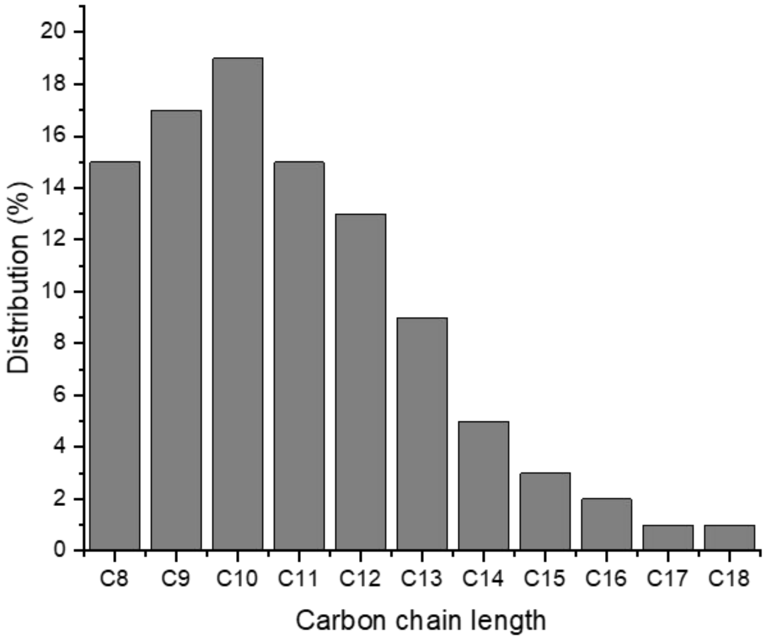

Supplementary Figure S2. DSC analysis of purified PHA produced by *P. resinovorans* using mixed alkane.

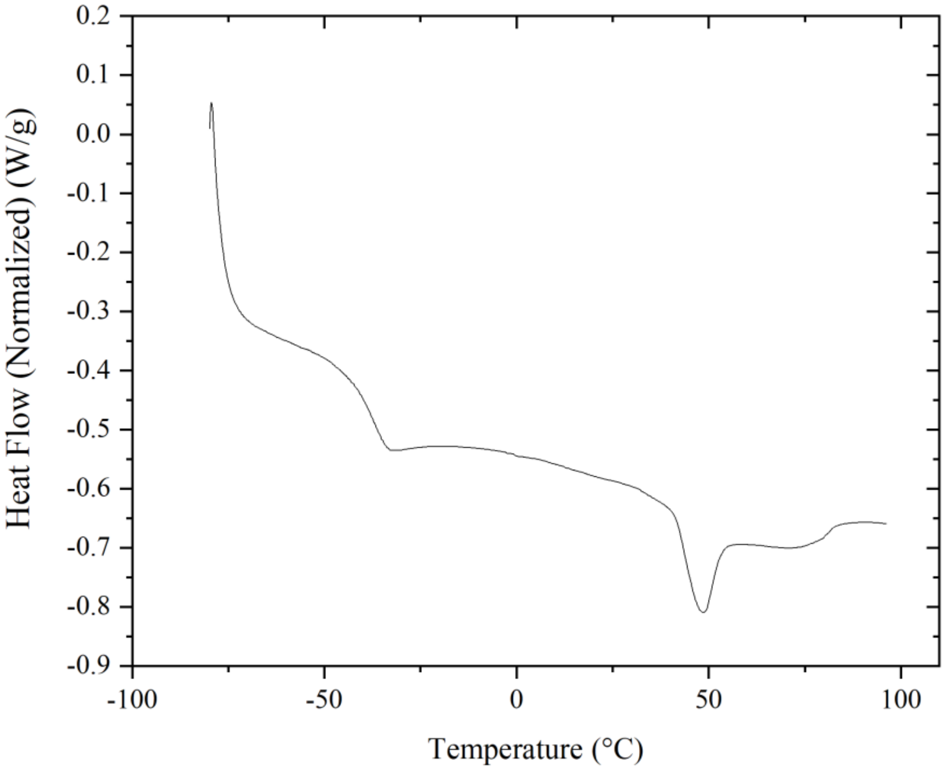

Supplement: Supplementary file 1 [file polymers-14-02624-s001.zip › polymers-1785788-supplementary.pdf]
